# Analysis of care needs for primary caregivers of premature infants at home based on the Kano model: A cross-sectional study

**DOI:** 10.1371/journal.pone.0360393

**Published:** 2026-10-08

**Authors:** Fenfen Lan, Jiaohua Yu, Zhaohua Huo, Yuanyuan Wang, Qiaoyun Shi, Yuanyu Liao, Juan Liu

**Affiliations:** 1 Department of Neonatology, Affiliated Central People’s Hospital, China Three Gorges University (Yichang Central People’s Hospital), Yichang, China; 2 Nursing Department, Union Hospital, Tongji Medical College, Huazhong University of Science and Technology, Wuhan, China; 3 Department of Emergency Medicine, Shenzhen Hospital of Southern Medical University, Shenzhen, China; Sichuan Provincial People's Hospital: Sichuan Academy of Medical Sciences and Sichuan People's Hospital, CHINA

## Abstract

**Objective:**

This study applied the Kano model to analyze the attributes of care needs among primary caregivers of discharged premature infants(up to 6 months post discharge), identify priority needs, and provide a foundation for developing targeted nursing service plans.

**Methods:**

Between November 2024 and January 2025, a cross-sectional survey was conducted using convenience sampling. Primary caregivers of premature infants discharged within the previous six months from a Grade A tertiary hospital in Hubei Province were recruited. A self-designed questionnaire based on the Kano model was administered to evaluate the attributes and priorities of caregivers’ needs. The questionnaire consisted of 27 care-need items. Kano attribute classification used the Maximum Frequency Method, and needs were prioritized using Better–Worse coefficients and the Average Satisfaction Coefficient (ASC).

**Results:**

A total of 201 valid questionnaires were collected. Analysis using the Kano model revealed that, among the 27 care needs, three were classified as “Must-be attributes” (related to infant feeding), six as “One-dimensional attributes” (related to health issues of premature infants), and 18 as “Attractive attributes.”.

**Conclusion:**

The care needs of primary caregivers of premature infants at home are generally high, with survival needs being the most urgent. Targeted support should be provided based on the priority of needs and attributes to optimize home care services and improve both the quality of care and health outcomes of premature infants.

## Introduction

Premature birth is a significant public health challenge worldwide, with an incidence rate of approximately 10%. In China, the preterm birth rate is approximately 6%, while the number of preterm births ranks fourth globally [1]. Preterm infants are susceptible to complications such as respiratory diseases, hyperbilirubinemia, feeding difficulties, and hypoglycemia, which often lead to prolonged hospitalization and markedly higher readmission rates [2]. Studies indicate that the readmission rate within two years after discharge can reach 56% among preterm infants, with late-preterm infants (34–36 weeks of gestation) facing twice the risk of readmission within 30 days compared to full-term infants [3].

Due to their organ immaturity, preterm infants often require admission to the Neonatal Intensive Care Unit (NICU), where they receive multidisciplinary professional care and continuous life support. Separation from their primary caregivers during hospitalization can disrupt the continuity of parent–infant bonding, impede the acquisition of care-related knowledge and skills, and delay the development of caregiving competence [4]. Consequently, families face multiple challenges after discharge, including insufficient caregiving capacity due to delayed adaptation to caregiving roles; anxiety and depression arising from uncertainties about the infant’s prognosis and development; fatigue caused by long-term, demanding care tasks; and isolation resulting from stigma or misconceptions [5].

In addition, primary caregivers are under considerable pressure to facilitate catch-up growth in preterm infants. The early postnatal period—particularly the first 6–12 months—represents a critical window for compensatory growth [6]. Providing scientifically informed and appropriate care during this crucial phase is essential for improving the quality of life for preterm infants. Therefore, post-discharge care is a long-term and complex process that requires caregivers to rapidly acquire the necessary competencies, imposing substantial demands and psychological pressure.

When confronted with health issues in preterm infants, primary caregivers—particularly those with limited coping capacity [7], psychological adjustment difficulties, or delayed role acceptance [8]—exhibit strong and diverse care needs. Research indicates that within six months after discharge, parents have multifaceted information needs aimed at improving their mental health and sleep quality, and they expect tailored resources and informational support [9]. The uncertainties and responsibilities inherent in caregiving often cause anxiety, underscoring the urgent need for informational and peer support to help caregivers navigate this transitional period [10,11]. Although caregivers acquire some basic skills through discharge education (such as bathing, dressing, and diaper changing), they frequently feel inadequately prepared for specialized care tasks, including home oxygen therapy, electrocardiogram monitoring, or nasogastric feeding. As infants progress through developmental stages, care demands continue to evolve, demonstrating both dynamism and complexity [12].

Accurate identification of caregivers’ needs is a prerequisite for providing effective support. At present, hospital and community nurses often struggle to prioritize these needs, resulting in generalized rather than targeted interventions. This mismatch between services and needs can reduce resource efficiency. Previous studies have shown that providing scientific feeding guidance alleviates feeding difficulties in preterm infants, while information on behavior, growth, and development enhances caregivers’ skills and confidence, reduces anxiety, and promotes parent-infant interaction [13]. However, the specific attributes and priorities of various needs remain unclear, limiting the precision of support. In addition, most existing research has focused on parental caregivers, with insufficient attention to the individualized needs of grandparents—who also serve as primary caregivers for some preterm infants [14]. The absence of population-specific analysis hinders the delivery of precisely matched services.

Since its introduction into nursing research, the Kano model has been widely applied to identify user needs, optimize service strategies, and promote innovation in nursing models and management practices [15]. Through a cross-sectional survey and Kano analysis, this study aims to clarify the attributes and priorities of care needs among primary caregivers of discharged preterm infants, thereby providing a foundation for developing targeted nursing interventions.

## Methods

### Study design and participants

A cross-sectional survey was conducted between November 1,2024 and January 31,2025 using convenience sampling. Primary caregivers of preterm infants discharged within the previous six months from a Grade A tertiary hospital in Hubei Province were recruited. The NICU of this hospital admits all preterm infants, mostly moderate-to-late preterm, and provides postoperative care for surgical conditions. The NICU operates a closed management system. During hospitalization, primary caregivers are not involved in daily care (e.g., feeding, bathing, diaper changing). At discharge, caregivers receive standardized health education on basic home care skills. This system is standard in most NICUs in China. The entire research design and reporting process followed the STROBE (Strengthening the Reporting of Observational Studies in Epidemiology) guidelines.

Inclusion criteria for preterm infants were: (1) gestational age < 37 weeks; and (2) discharge within the past 6 months. Exclusion criteria for preterm infants were: (1) severe complications including moderate-to-severe anemia, severe retinopathy of prematurity requiring treatment, severe intraventricular hemorrhage, necrotizing enterocolitis requiring surgery, and moderate-to-severe bronchopulmonary dysplasia; (2) genetic disorders or congenital malformations; (3) transfer to another hospital due to medical needs; and (4) discharge against medical advice at the family’s request.

Inclusion criteria for primary caregivers were: (1) adult primary caregivers, female ≥20 years old, male ≥22 years old [16]; (2) a family member (parent or grandparent) serving as the main caregiver with ≥ 6 h of daily caregiving; (3) normal cognitive function and communication ability; and (4) voluntary participation in the survey. Exclusion criteria for primary caregivers were: (1) caregivers whose caregiving ability was impaired by severe cardiac, cerebral, pulmonary, or renal diseases; and (2) withdrawal during the study.

Note on participant age: All primary caregiver participants met the inclusion criterion of being adults (female ≥20 years, male ≥22 years). Therefore, no minors were included as direct participants in this survey, and the requirement for parental or guardian consent was not applicable.

### Questionnaire survey

#### Demographic questionnaire.

A self-designed form was used to collect demographic information, including infants’ gestational age, birth weight, singleton status, and caregivers’ age, gender, relationship to the infant, education level, caregiving experience, and place of residence.

#### Home care needs questionnaire for primary caregivers of preterm infants.

The care needs questionnaire was developed through a systematic process:

First, keywords such as “preterm infant”, “post-discharge”, “home care”, “primary caregiver”, and “needs” were used to search Chinese and English databases (PubMed, Web of Science, Embase, Cochrane, CINAHL, CNKI, Wanfang, VIP, CBM). High-frequency care needs were extracted and categorized based on the ERG(Existence, Relatedness, and Growth) needs theory, and an initial draft was prepared through research team discussions.

Next, qualitative interviews were conducted with 25 eligible primary caregivers to identify practical service needs during home care.

Finally, 21 clinical nursing experts with over 10 years of experience, at least a bachelor’s degree, and intermediate or senior professional titles(nurse-in-charge or above, including associate chief nurse and chief nurse) were invited to review and revise the questionnaire, resulting in a preliminary version.

Following the principles of the Kano model, each item was phrased in both positive and negative forms. A pretest was then conducted among primary caregivers who met the inclusion criteria, in which participants were asked to review the questionnaire items and provide feedback on any unclear or difficult-to-understand wording. Items were revised based on their feedback until all participants found the questionnaire easy to understand. This pretest was conducted to assess the feasibility of the questionnaire. The formal survey was administered to primary caregivers of preterm infants who had been discharged within the previous six months. The internal consistency (Cronbach’s α) was 0.932 for the positive version and 0.940 for the negative version, demonstrating strong reliability. Exploratory factor analysis retained 27 items across three dimensions: Existence needs, Relatedness needs, and Growth needs. Confirmatory factor analysis showed that the model fit indices of both versions met acceptable standards, with good convergent and discriminant validity. The item-level content validity index (I-CVI) ranged from 0.71 to 1.00, and the scale-level content validity index (S-CVI) was 0.93, indicating satisfactory validity. The final questionnaire consisted of 27 items across three dimensions: 16 in Existence needs, 7 in Relatedness needs, and 4 in Growth needs (S1 File).

### Kano model

#### Quality attributes.

The Kano model, developed by Professor Noriaki Kano and inspired by Herzberg’s Two-Factor Theory, is used to analyze how product or service attributes affect user satisfaction [17]. The model classifies quality attributes into five categories: Must-be (M), One-dimensional (O), Attractive (A), Indifferent (I), and Reverse (R).

#### Kano questionnaire.

A specific Kano questionnaire format was employed, presenting each service item in both positive and negative forms. For example: Positive question: “How would you feel if this service were provided?”

Negative question: “How would you feel if this service were not provided?” Each question offered five response options: “Like,” “Must-be,” “Neutral,” “Live with,” and “Dislike”.

#### Kano attribute analysis.

The attribute category for each need was determined by cross-referencing responses to the positive and negative questions using the evaluation matrix The final attribute for each item was identified using the “Maximum Frequency Method”.

#### Prioritization of needs.

To address limitations of the traditional Kano model in distinguishing priorities when attribute frequencies were similar, the Better–Worse coefficient proposed by Berger et al. [18] was applied for quantitative analysis: Satisfaction index (Better): (A + O)/ (A + O + M + I); Dissatisfaction index (|Worse|): |(O + M)/ (A + O + M + I)|. Needs were prioritized by quadrant in the following order: Quadrant IV > I > II > III. To further rank needs within the same quadrant, the Average Satisfaction Coefficient (ASC) [19] was applied: ASC = (Better + |Worse|)/ 2. A higher ASC indicates a higher priority. When ASC values were equal, the item with a higher absolute Worse value was prioritized.

### Sample size calculation

Based on Kendall’s guideline for sample size estimation in cross-sectional studies, the required sample size should be 5–10 times the number of questionnaire items [20]. Considering a 10% rate of invalid responses, the sample size was calculated as N = 27 × (5–10) ÷ 0.9 ≈ 150–300.

### Data collection and analysis

#### Data collection.

With approval from the medical institution, paper-based questionnaires were distributed at follow-up clinics. To ensure data quality, home visits were conducted for caregivers who were unable to complete the questionnaire during clinic visits because of childcare responsibilities or missed appointments, provided that they agreed to participate.

#### Data analysis.

Data were independently entered into EpiData 3.1 by two researchers, followed by cross-verification to ensure accuracy, and analyzed with SPSS 26.0. Categorical data were summarized using frequencies and proportions. Kano attributes were classified according to the Maximum Frequency Method. A two-dimensional matrix was constructed using Better–Worse coefficients, and needs were prioritized according to ASC values.

#### Quality control.

All researchers received standardized training and conducted pilot surveys to become familiar with the procedures. Participants were informed that participation was voluntary, anonymous, and confidential before participation. For respondents with lower education levels or advanced age, researchers read each item aloud and recorded their responses. Questionnaires were reviewed on-site for completeness and accuracy. Data were double-entered and cross-verified to ensure data integrity.

### Ethical considerations

This study was approved by the Medical Ethics Committee of Tongji Medical College, Huazhong University of Science and Technology (Approval Number: [2023] Lun Shen Zi (S134)) for the initial research protocol. The implementation of the cross-sectional survey at the primary data collection site was further reviewed and approved by the Medical Ethics Committee of Yichang Central People’s Hospital (Approval Number: 2024-311-01). The latter committee explicitly granted a waiver of written informed consent, considering the study to be an anonymous, low-risk survey. All procedures were conducted in accordance with the ethical principles of the Declaration of Helsinki.

## Results

### Participant characteristics

During the survey period (November 1, 2024 to January 31, 2025), a total of 125 preterm infants discharged from the study hospital met the inclusion criteria (gestational age < 37 weeks, discharged within the previous 6 months). Ultimately, 98 preterm infants were included in the final analysis. A total of 220 questionnaires were distributed, and 201 valid responses were collected, resulting in an effective response rate of 91.36%. Valid responses were defined as questionnaires with no missing data on the 27 Kano items and no contradictory responses (e.g., “Like” for both positive and negative forms of the same item). The general characteristics of the included preterm infants (n = 98) and their primary caregivers (n = 201) are summarized in Table 1.

**Table 1:** General characteristics of preterm infants and their primary caregivers (N = 201).

Preterm infants				Primary caregivers			
Category	Group	n	%	Category	Group	n	%
Gender	Female	41	41.84	Gender	Female	145	72.14
	Male	57	58.16		Male	56	27.86
				Age (years)	< 35	105	52.24
					35–59	43	21.39
Singleton	Yes	56	57.14		≥ 60	53	26.37
	No	42	42.86	Relationship	Mother	72	35.82
					Father	50	24.88
Gestational age (weeks)	28–31	6	6.12		Grandmother	43	21.39
	32–33	43	43.88		Grandfather	6	2.99
	34–37	49	50.00		Maternal grandmother	30	14.93
				Academic qualification	Primary school or below	12	5.97
					Junior high school	52	25.87
					High school/ vocational school	33	16.42
Birth weight (kg)	1–1.4	3	3.06		College degree	52	25.87
	1.5–2.4	92	93.88		Bachelor’s degree or above	52	25.87
	≥ 2.5	3	3.06	Parenting experience	Yes	93	46.27
					No	108	53.73
				Occupation	Government/ public institution	26	12.94
Time since discharge (months)	< 1	17	17.35		Enterprise employee	65	32.34
	1- < 2	10	10.20		Freelancer	43	21.39
	2– < 3	17	17.35		Farmer/ laborer	4	1.99
	3– < 4	27	27.55		Unemployed/ other	63	31.34
	4– < 5	15	15.31	Residence	Urban area	177	88.06
	5–6	12	12.24		Non-urban areas	24	11.94

### Classification of care needs attributes

Based on the maximum frequency method, the 27 care needs were classified into three categories: three were Must-be attributes, six were One-dimensional attributes, and the remaining 18 were Attractive attributes.

#### Must-be attributes.

These are basic services that caregivers expect to be provided; their absence significantly reduces satisfaction. The Must-be attributes included: “Informing the timing and method of formula transition,” “Informing medication usage and related precautions,” and “Guiding the assessment of feeding timing and amount.”

#### One-dimensional attributes.

These are services for which provision is linearly correlated with satisfaction. The six identified One-dimensional attributes were: “Providing knowledge about growth and development patterns,” “Guiding early intellectual care training for preterm infants,” “Guiding identification of abdominal distension,” “Guiding identification of abnormal jaundice,” “Guiding management of vomiting and choking,” and “Guiding first aid for choking and suffocation.”

#### Attractive attributes.

These are services that exceed expectations and significantly enhance satisfaction. A total of 18 needs were classified into this category, including “Educating about normal urination and defecation,” “Teaching techniques to promote intestinal gas relief,” and “Guiding maintenance for oxygen therapy or tube feeding at home.” Detailed results are presented in Table 2.

**Table 2:** Summary of Kano model analysis results.

Function/ Service	A	O	M	I	Classification
1. Informing caregivers about the timing and method of formula transition (from special formula to regular formula or breast milk)	59	42	68	32	M
2. Informing caregivers about medication usage and related precautions	41	59	92	9	M
3. Providing caregivers with knowledge about growth and development patterns	63	118	12	8	O
4. Educating caregivers about normal urination and defecation	109	65	10	17	A
5. Guiding caregivers in assessment of feeding timing and amount	58	50	76	17	M
6. Instructing caregivers on early intellectual care training (e.g., visual/auditory sensory stimulation, passive exercise)	55	127	9	10	O
7. Teaching caregivers techniques to promote intestinal gas relief	109	77	5	10	A
8. Guiding caregivers in maintaining oxygen or tube feeding at home	75	17	45	64	A
9. Instructing caregivers on identifying abdominal distension	54	125	5	17	O
10. Guiding caregivers in recognizing and managing defecation (diarrhea or constipation)	79	46	28	48	A
11. Teaching caregivers how to identify abnormal jaundice	61	77	48	15	O
12. Instructing caregivers on managing vomiting and choking during feeding	62	65	60	14	O
13. Training caregivers in first aid for accidental falls from the bed	111	67	9	14	A
14. Training caregivers in first aid for choking and suffocation	59	121	8	13	O
15. Demonstrating infant massage techniques to caregivers	140	29	7	25	A
16. Teaching caregivers eye contact and vocal interaction skills	114	55	9	23	A
17. Providing caregivers with diversified hospital consultation methods (e.g., online, in-person accompaniment, special appointments)	123	50	7	21	A
18. Disseminating disease-related information through science lectures, official social media, and online platforms	130	43	5	23	A
19. Organizing multiple follow-up formats (outpatient visits, home visits, telephone calls)	129	44	9	19	A
20. Providing caregivers with information on financial aid and access channels	107	56	12	26	A
21. Facilitating peer support among caregivers of preterm infants	122	57	3	19	A
22. Enabling assistance from family members	145	37	2	17	A
23. Promoting shared decision-making within the family	122	55	2	22	A
24. Supporting caregivers’ emotional stability	129	42	4	26	A
25. Encouraging caregivers to independently care for the preterm infant	98	52	22	29	A
26. Offering training workshops to improve caregivers’ skills	131	45	3	22	A
27. Creating platforms for sharing successful care experiences among caregivers	131	43	4	23	A

Legend: A = Attractive attribute; O = One-dimensional attribute; M = Must-be attribute; I = Indifferent attribute.

### Prioritization of care needs

#### Better–Worse coefficient analysis.

The Better–Worse coefficients were calculated to further analyze the prioritization of needs (results are presented in Table 3). The highest Better value was observed for “Teaching caregivers techniques to promote intestinal gas relief” (0.925), indicating that providing this service would increase satisfaction by 92.5%. The highest absolute Worse value was observed for “Informing caregivers about medication usage and related precautions” (0.751), indicating that not providing this service would decrease satisfaction by 75.1%.

**Table 3:** Better–Worse coefficients and ASC values.

Function/ Service	Better	Worse (absolute)	ASC
1. Informing caregivers about the timing and method of formula transition (from special formula to regular formula, or breast milk)	0.503	0.547	0.525
2. Informing caregivers about medication usage and related precautions	0.498	0.751	0.624
3. Providing caregivers with knowledge about growth and development patterns	0.901	0.647	0.774
4. Educating caregivers about normal urination and defecation	0.866	0.373	0.619
5. Guiding caregivers in assessing feeding timing and amount	0.537	0.627	0.582
6. Instructing caregivers on early intelligence care training (e.g., visual/auditory sensory stimulation, passive exercise)	0.906	0.677	0.791
7. Teaching caregivers techniques to promote intestinal gas relief	0.925	0.408	0.667
8. Guiding caregivers in maintenance oxygen therapy or tube feeding at home	0.458	0.309	0.383
9. Instructing caregivers on identifying abdominal distension	0.891	0.647	0.769
10. Guiding caregivers in recognizing and managing abnormal defecation (diarrhea or constipation)	0.687	0.368	0.527
11. Teaching caregivers how to identify abnormal jaundice	0.622	0.622	0.622
12. Instructing caregivers on managing vomiting and choking during feeding	0.632	0.622	0.627
13. Training caregivers in first aid for accidental falls from bed	0.886	0.378	0.632
14. Training caregivers in first aid for choking and suffocation	0.896	0.642	0.769
15. Demonstrating infant massage techniques to caregivers	0.841	0.179	0.510
16. Teaching caregivers eye contact and vocal interaction skills	0.841	0.318	0.580
17. Providing caregivers with diversified hospital consultation methods (e.g., online, in-person accompaniment, special appointments)	0.861	0.284	0.572
18. Disseminating disease-related information through science lectures, official social media, and online platforms	0.861	0.239	0.550
19. Organizing multiple follow-up formats (outpatient visits, home visits, telephone calls)	0.861	0.264	0.562
20. Providing caregivers with information on financial aid and access channels	0.811	0.338	0.575
21. Facilitating peer support among caregivers of preterm infants	0.891	0.299	0.595
22. Enabling assistance from family members	0.906	0.194	0.550
23. Promoting shared decision-making within the family	0.881	0.284	0.582
24. Supporting caregivers’ emotional stability	0.851	0.229	0.540
25. Encouraging caregivers to independently care of the preterm infant	0.746	0.368	0.557
26. Offering training workshops to improve caregivers’ skills	0.876	0.239	0.557
27. Creating platforms for sharing successful care experiences among caregivers	0.866	0.234	0.550

#### Needs prioritization.

A two-dimensional matrix was constructed based on the Better–Worse coefficients (Fig 1), with the mean Better and absolute Worse values used as the origin to divide the matrix into four quadrants. Needs were prioritized by quadrant in the following order: Quadrant IV > I > II > III. Further ranking within each quadrant was performed using the Average Satisfaction Coefficient (ASC), as presented in Table 4, resulting in a hierarchical model of care needs priorities for primary caregivers of discharged preterm infants.

**Table 4:** Quadrant distribution and priority ranking of care needs for primary caregivers of discharged preterm infants.

quadrant	Item	Priority Rank
Quadrant IV (To-be-Improved)	Guiding caregivers in managing vomiting and choking	1
	Informing caregivers about medication usage and related precautions	2
	Guiding caregivers in identifying abnormal jaundice	3
	Guiding caregivers in assessing feeding timing and amount	4
	Informing caregivers about the timing and method of formula transition from special formula to regular formula or breast milk	5
Quadrant I (Advantage)	Instructing caregivers on early intellectual care training (e.g., visual/auditory sensory stimulation, passive exercise)	6
	Providing caregivers with knowledge about growth and development patterns	7
	Guiding caregivers in identifying abdominal distension	8
	Training caregivers in first aid for choking and suffocation	9
Quadrant II (Maintenance)	Training caregivers in first aid for accidental falls from the bed	10
	Teaching caregivers techniques to promote intestinal gas relief	11
	Educating caregivers about normal urination and defecation	12
	Facilitating peer support among caregivers of preterm infants	13
	Promoting shared decision-making within the family	14
	Teaching caregivers eye contact and vocal interaction skills	15
	Providing caregivers with information on financial aid and access channels	16
	Providing caregivers with diversified hospital consultation methods (e.g., online, in-person accompaniment, special appointments)	17
	Organizing multiple follow-up formats (outpatient visits, home visits, telephone calls)	18
	Offering training workshops to improve caregivers’ skills	19
	Creating platforms for sharing successful care experiences among caregivers	20
	Enabling assistance from family members	21
	Disseminating disease-related information through science lectures, official social media, and online platforms	22
	Supporting caregivers’ emotional stability	23
	Demonstrating infant massage techniques	24
Quadrant III (Low Priority)	Encouraging caregivers to independently care of the preterm infant	25
	Guiding caregivers in recognizing and managing abnormal defecation (diarrhea or constipation)	26
	Guiding caregivers in maintaining oxygen therapy or tube feeding at home	27

**Fig 1:**
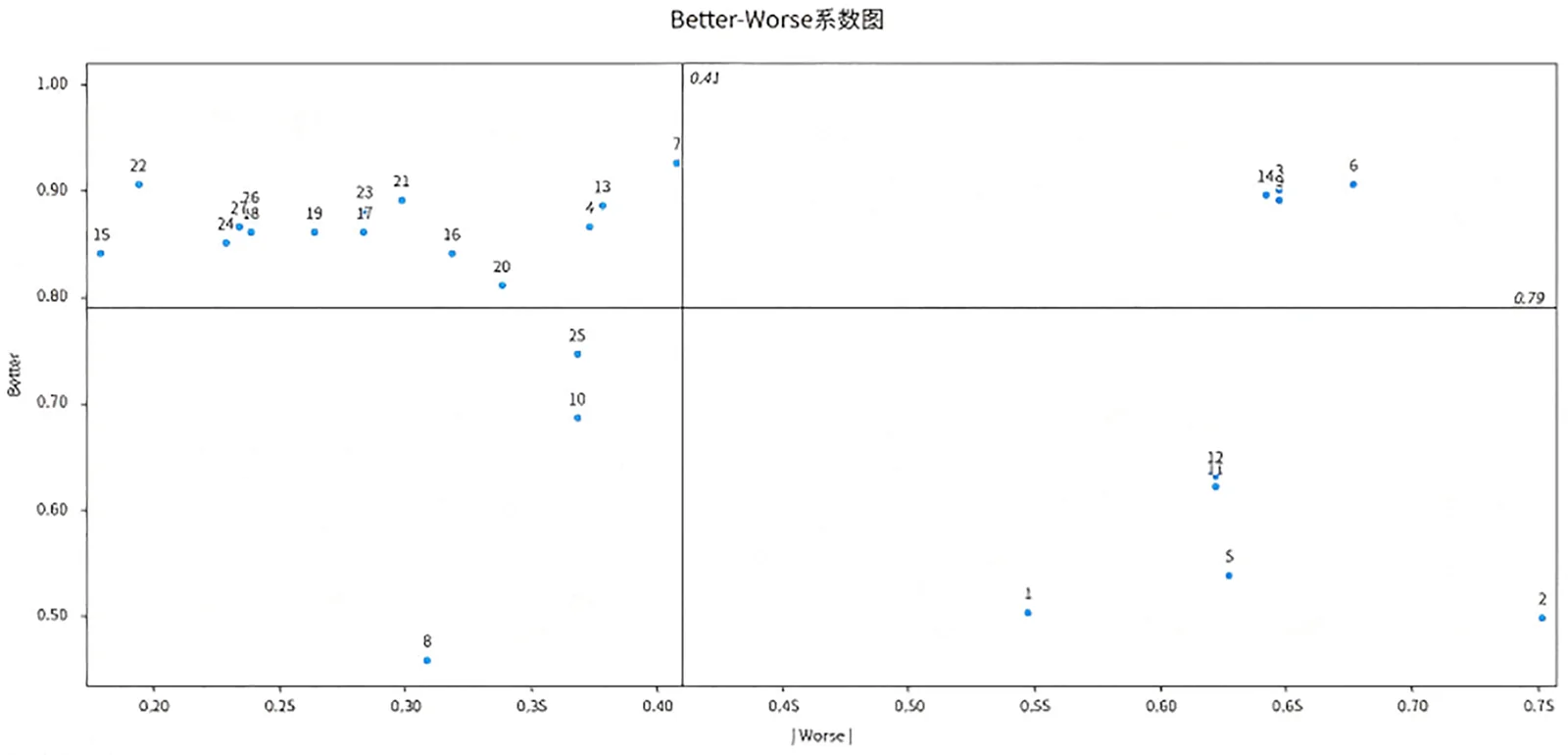
Better–Worse coefficient matrix.

## Discussion

### High level of care needs among primary caregivers of discharged preterm infants

The analysis of 27 care needs using the Maximum Frequency Method revealed three Must-be attributes, six One-dimensional attributes, and 18 Attractive attributes, indicating that primary caregivers have extensive and complex need during home care. Beyond daily care knowledge, caregivers urgently require professional, social, and family support, and are particularly concerned with improving their caregiving competencies. This finding is consistent with a recent qualitative study of mothers of preterm infants [21], which highlighted that maternal caregiving is a multidimensional concept involving cognitive, technical, and psychological dimensions. As non-professional caregivers, mothers’ strong sense of responsibility and concern for their infants’ health lead them to have expectations for nursing services.

The closed management model adopted by most NICUs in China may explain the caregivers’ insufficient caregiving capacity after discharge, as it prevents them from participating in care immediately after birth. During home care, the knowledge caregivers acquire from discharge education is limited. Moreover, as preterm infants grow, new health issues emerge that require continuous access to professional information. However, existing community maternal and child health services are often inadequate to meet these needs. Consequently, in addition to clinical follow-up, most caregivers turn to the internet for information [22] Unfortunately, the variable quality of online information limits caregivers’ access to reliable care knowledge [23]. To address the above issues, the following measures are recommended: develop and promote additional support tools to help caregivers monitor infant growth and intervene in a timely manner; strengthen health education and support services to enhance family health management capacity; and provide psychological counseling to alleviate caregiving stress, thereby improving caregivers’ mental health and caregiving ability.

Our findings are generally consistent with the direction of several recent studies. Lebel et al. [9] showed that parents of preterm infants have multifaceted information needs during the first six months after discharge, and meeting these needs may help improve their mental health and sleep quality. Similarly, Flierman et al. [22] found that parents require continuous access to professional information as their preterm infant grows, which aligns with our observation that caregivers’ needs may extend beyond basic discharge education. Building on these studies, the present study attempted to quantify the relative importance of different needs using the Kano model. Our preliminary results suggest that although various needs may all be important to some extent, survival-related needs appear to receive greater attention from caregivers.

### Survival needs are the most urgent requirements for caregivers

The results show that the six One-dimensional and three Must-be attributes all pertain to survival needs, concentrated in Quadrants I and IV, underscoring the high-risk nature of preterm infants. Complications arising from organ immaturity—such as choking, suffocation, and pathological jaundice—directly threaten infant survival, prompting caregivers to prioritize basic life-sustaining safeguards.

Feeding-related needs (e.g., assessing feeding timing and amount and determining transition timing and methods) were identified as Must-be attributes with high priority, consistent with previous research. For instance, da Cunha et al. [24] reported that feeding was the most frequently reported need among caregivers of preterm infants. Building on this, the present study further classifies feeding needs as Must-be attributes, suggesting that caregivers regard feeding support as a basic expectation, and that a lack of such services may lead to significant dissatisfaction. This classification has clear practical implications: healthcare providers should prioritize feeding support as an essential service rather than an optional one. Moreover, da Cunha’s study also found that, unlike caregivers of full-term infants, the feeding needs of preterm infant caregivers remain high over an extended period.

Notably, life-saving skill guidance such as choking first aid was not classified as a Must-be attribute in this study. This phenomenon may be related to caregivers’ psychological factors: on the one hand, parents generally have low self-efficacy regarding their own first-aid abilities; on the other hand, although such events have severe consequences, their low frequency may lead to cognitive biases, causing caregivers to underestimate the necessity of related guidance.

Furthermore, the classification of first-aid skill needs may also be influenced by healthcare systems and sociocultural contexts. Existing research indicates that the accessibility of infant cardiopulmonary resuscitation (CPR) education varies significantly across regions, populations, and healthcare systems. For example, a systematic review noted that infant CPR is generally not integrated into mainstream perinatal parenting education programs; even in American neonatal intensive care units (NICUs) that require CPR training prior to discharge, only about 37% of parents actually completed the training, and accessibility is even more limited in resource-poor settings (e.g., Indigenous communities in Australia) [25]. Further studies have found that the provision rate of bystander CPR also differs significantly across socioeconomic groups [26].

These findings suggest that caregivers’ perceptions of first-aid skill needs are closely related to their specific healthcare environment, cultural background, and socioeconomic status. Therefore, context-specific needs assessment has important practical implications: interventions developed in one healthcare system should be adapted to local circumstances rather than being directly transplanted.

Needs closely related to health—such as understanding growth patterns, intellectual care training, choking first aid, and identifying abnormal jaundice—were all highly One-dimensional, indicating that feeding and health monitoring are core concerns. This pattern may be related to the high proportion of grandparents and fathers in this study. Research shows [27,28] that parents of preterm infants often rely on grandparents’ experiential knowledge during the early post-discharge period. Grandparents typically depend on traditional parenting experience, are reluctant to seek professional help, and focus primarily on feeding and weight. Fathers, on the other hand, often have limited involvement, delayed role adaptation, and low confidence [29], tending to focus more on physical indicators such as infant weight [30].

Therefore, in continuing feeding care, “cue-based feeding strategies” should be promoted. These strategies combine scheduled feeding with responsiveness to infant hunger and satiety cues to improve feeding success and support appropriate growth [31]. Caregivers need to accurately identify feeding cues (e.g., the infant waking up and searching for the nipple) and stopping cues (e.g., falling asleep or stopping sucking), and adopt a “semi-demand feeding” model after discharge. This model integrates the infant’s responses with time and volume settings to scientifically assess feeding timing and amount. This approach can enhance caregiving capacity and reduce readmission rates.

It is advisable to include formula transition knowledge in routine health education and to disseminate related information through professional channels such as hospital social media accounts, internet-based nursing clinics, and preterm infant support groups. This would help caregivers achieve evidence-based formula transition and rational nutrient management.

For intellectual care training, caregivers should be guided at discharge to implement interventions aligned with developmental milestones (e.g., rolling over, sitting, crawling, standing, and walking). They should also be taught methods such as touch, passive exercise, and tactile stimulation to promote physical, motor, and intellectual development.

Aspiration suffocation and accidental bed suffocation are the two most common types of infant suffocation [32]. In China, these events are closely associated with bed-sharing practices and inadequate care, and without prompt intervention, they can result in death [33,34]. Therefore, training on prevention and first aid should be strengthened for caregivers of preterm infants, delivering professional knowledge and practical skills in an accessible and acceptable way.

Readmission due to hyperbilirubinemia is often linked to breastfeeding problems [35]. It is recommended that follow-up plans be developed at discharge based on bilirubin levels and risk factors, and that caregivers be guided to recognize abnormal jaundice and seek timely medical attention to reduce the harm caused by pathological jaundice and lower readmission rates.

### Implications of needs prioritization for clinical nursing management

#### Constructing a survival needs support system.

The prioritization of survival needs aligns with the “proximal needs first” principle in Bronfenbrenner’s ecological systems theory. In the future, a “Home Care First-Aid Kit for Preterm Infants” and standardized training programs could be developed, incorporating simulated choking management exercises. A digital medication management assistant could provide personalized medication guidance, and stepped feeding plans could be developed alongside intelligent assessment tools integrated with growth charts to systematically build a survival needs support system.

#### Optimizing the support system and establishing a “Hospital–Community-Family” tertiary linkage mechanism.

The high survival needs in home-based care for preterm infants reflect both biomedical vulnerability and inadequacies in the social support system. It is recommended to establish a multi-agency collaborative “hospital–community–family” tertiary linkage mechanism [36], implement a family-centered care model, and enhance caregiver participation and satisfaction [37]. Specific measures include: allowing parents to enter the NICU during hospitalization to learn non-medical care; providing post-discharge services such as home visits and online guidance from multidisciplinary teams; organizing regular online and offline follow-ups by community nurses to assess development and provide long-term guidance, with timely referrals for infants with developmental delays [38,39];and establishing a cloud-based parent mutual support platform through hospitals and communities to facilitate experience sharing and stress relief, alleviating relational pressures, and ultimately transitioning from passive receipt of care guidance to autonomous caregiving.

In summary, the high level of needs among primary caregivers of preterm infants reflects rising caregiving expectations amid social development. There is a need to strengthen survival support through dynamic response mechanisms, improve home care quality, and reduce avoidable readmission risks. Future efforts should further integrate technology, resources, and policies to establish a needs-oriented precision support system, transforming “high expectations” into “high satisfaction” and ultimately achieving family-centered whole-cycle health management for preterm infants.

### What this study adds to existing research

This study may offer several potential contributions to the existing literature. First, this study applies the Kano model—traditionally used in marketing and service design—to assess the home care needs of primary caregivers of preterm infants, providing a possible methodological reference for nursing research. Second, unlike previous studies that have primarily listed caregiver needs, this study classifies them into Must-be, One-dimensional, and Attractive attributes using the Maximum Frequency Method, which may offer a useful reference for prioritizing needs in resource-limited clinical settings. Third, by including grandparents and fathers as primary caregivers, this study supplements the perspectives of previous research that has focused predominantly on mothers. Fourth, the Better–Worse coefficient analysis may provide quantifiable indicators for service design; for example, the satisfaction coefficient for teaching techniques to promote intestinal gas relief was 0.925 in this study, suggesting that such services may have high potential to improve caregiver satisfaction. These preliminary findings may help clinicians and nurses prioritize the most urgent survival needs of preterm infant caregivers while also exploring attractive services that could enhance long-term satisfaction.

### Limitations

This study has several limitations. First, the sample consisted predominantly of late preterm infants, which may have introduced bias. As a single-center investigation, the findings may not fully represent all primary caregivers of discharged preterm infants due to regional and cultural constraints. Second, the sample size of extremely and very preterm infants (<32 weeks) was only 6 cases (6% of the total sample), which precluded stable subgroup analysis by gestational age. Consequently, potential differences in caregiver needs across different levels of prematurity could not be adequately examined. Third, this cross-sectional study captured caregiver needs at a single time point, and the sample distribution across different post-discharge periods was uneven. This limited our ability to perform stable subgroup analysis by time since discharge, as caregiver needs may evolve over time with infant growth. Future research could build on this study by conducting multicenter, large-sample, longitudinal studies to examine the diverse care needs of primary caregivers over time. Dynamically tracking changes in need priorities and exploring variations across different caregiver profiles would provide an empirical basis for developing more targeted and timely care support strategies. Nevertheless, this study provides a valuable methodological approach for identifying and addressing the care needs of this population.

## Conclusion

This cross-sectional study applied the Kano model to analyze the home care needs of primary caregivers of preterm infants. The results showed that primary caregivers generally have high levels of care needs, among which survival-related needs (e.g., feeding, medication management, and choking prevention) are the most urgent. The Kano model provides a practical analytical framework for classifying and prioritizing these needs.

For clinical practice, Must-be and One-dimensional needs (e.g., feeding guidance, identification of jaundice) should be prioritized as essential services, while Attractive needs (e.g., infant massage instruction, peer support) can be gradually developed to enhance long-term satisfaction with care. The hierarchical needs priority model developed in this study may help clinicians allocate resources appropriately when caregivers have multiple competing needs. For researchers, the Kano model proved to be a valid tool for needs classification and prioritization in this context.

Future research could adopt multicenter longitudinal designs to track the dynamic changes in caregiver needs as preterm infants grow. Intervention studies could also be conducted to further validate the clinical effectiveness of service models based on this needs-priority framework.

## Supporting information

S1 Data The data. (XLS)

S1 File The Kano questionnaire on care needs of primary caregivers of premature infants. (DOCX)
