# Supplementary material for: Analysis of care needs for primary caregivers of premature infants at home based on the Kano model: A cross-sectional study
Source: PLoS One. 2026 Oct 8;21(10):e0360393. doi: 10.1371/journal.pone.0360393 (PMC13648993; doi:10.1371/journal.pone.0360393)
Supplement: Supplementary file 1 [file pone.0360393.s002.docx]

****Dear Caregivers,****

Hello! We are a research team from the Department of Neonatology, Yichang Central People's Hospital. We are conducting a survey on the home care needs of primary caregivers of premature infants after discharge. To help you better care for your baby, and to enable the hospital and community to provide you with more targeted nursing support, we hope to understand your real needs during the home care process of your premature infant (including nursing knowledge, skill guidance, psychological support, etc.).

Participation in this survey is entirely voluntary. You may choose whether to participate or not, and you may withdraw at any time without any impact on your or your baby's medical care. Your personal information will be kept strictly confidential. The information you provide will only be used for research purposes and will not be disclosed to any individuals or organizations other than the research team. We will keep your responses confidential and sincerely hope for your support.

Please read each question carefully, answer honestly according to your actual situation, and return the completed questionnaire to the investigator.

Thank you for your support and cooperation!

## **Part One: Sample Characteristics**

### **I. General Information of Preterm Infant**

1.Gender: □ Male □ Female

2.Singleton: □ Yes □ No

3.Gestational age: ______ weeks

4.Birth weight: ______ kg

5.Discharge date: ______ year ______ month

### **II General Information of Primary Caregiver**

1.Gender: □ Male □ Female

2.Relationship to infant: □ Father □ Mother □ Paternal grandmother □ Paternal grandfather □ Maternal grandmother □ Maternal grandfather

3.Age: ______ years

4.Education level: □ Primary school or below □ Junior high school □ High school or vocational school □ Associate degree □ Bachelor's degree or above

5.Occupation: □ Government/Public institution □ Enterprise □ Freelancer □ Farmer/Laborer □ Unemployed/Other

6.Previous parenting experience: □ Yes □ No

7.Residence: □ Urban area □ Non-urban area

**Part Two: Formal Scale**

****Instruction:**** This questionnaire aims to understand your actual care needs during the process of caring for your premature infant. Please rate your feelings about the provision or non-provision of each service. Please tick (√) the option that best reflects your feelings.

5-Like

4-Must-be

3-Neutral

2-Live with

1-Dislike

| Item | If provided | If NOT provided |
| --- | --- | --- |
| **1. Informing about the timing and method of formula transition (e.g., between special formula, regular formula, and breast milk)** | 5 4 3 2 1 | 5 4 3 2 1 |
| **2. Informing about medication usage and related precautions** | 5 4 3 2 1 | 5 4 3 2 1 |
| **3. Providing knowledge about growth and development patterns** | 5 4 3 2 1 | 5 4 3 2 1 |
| **4. Educating about normal urination and defecation** | 5 4 3 2 1 | 5 4 3 2 1 |
| **5. Guiding assessment of feeding timing and amount** | 5 4 3 2 1 | 5 4 3 2 1 |
| **6. Guiding early intellectual care training (e.g., visual/auditory sensory stimulation, passive exercise)** | 5 4 3 2 1 | 5 4 3 2 1 |
| **7. Teaching techniques to promote intestinal gas relief** | 5 4 3 2 1 | 5 4 3 2 1 |
| **8. Guiding maintenance of home oxygen therapy or tube feeding** | 5 4 3 2 1 | 5 4 3 2 1 |
| **9. Guiding identification of abdominal distension** | 5 4 3 2 1 | 5 4 3 2 1 |
| **10. Guiding identification and management of abnormal defecation (diarrhea, constipation)** | 5 4 3 2 1 | 5 4 3 2 1 |
| **11. Guiding identification of abnormal jaundice** | 5 4 3 2 1 | 5 4 3 2 1 |
| **12. Guiding management of vomiting and choking during feeding** | 5 4 3 2 1 | 5 4 3 2 1 |
| **13. Guiding first aid for accidental falls from bed** | 5 4 3 2 1 | 5 4 3 2 1 |
| **14. Guiding first aid for choking and suffocation** | 5 4 3 2 1 | 5 4 3 2 1 |
| **15. Demonstrating infant massage techniques** | 5 4 3 2 1 | 5 4 3 2 1 |
| **16. Teaching eye contact and vocal interaction skills** | 5 4 3 2 1 | 5 4 3 2 1 |
| **17. Hospital providing diverse consultation methods (online consultation, in-person accompaniment, special appointments, etc.)** | 5 4 3 2 1 | 5 4 3 2 1 |
| **18. Hospital providing disease-related information through science lectures, official social media, and online platforms** | 5 4 3 2 1 | 5 4 3 2 1 |
| **19. Hospital providing multiple follow-up formats (outpatient visits, home visits, telephone calls, etc.)** | 5 4 3 2 1 | 5 4 3 2 1 |
| **20. Providing information on financial aid and access channels** | 5 4 3 2 1 | 5 4 3 2 1 |
| **21. Access to peer support (assistance from other preterm infant caregivers)** | 5 4 3 2 1 | 5 4 3 2 1 |
| **22. Receiving help from family members** | 5 4 3 2 1 | 5 4 3 2 1 |
| **23. Making shared decisions with family members about infant care** | 5 4 3 2 1 | 5 4 3 2 1 |
| **24. Maintaining emotional stability during caregiving** | 5 4 3 2 1 | 5 4 3 2 1 |
| **25. Caring for the preterm infant independently** | 5 4 3 2 1 | 5 4 3 2 1 |
| **26. Participating in training workshops to improve caregiving skills** | 5 4 3 2 1 | 5 4 3 2 1 |
| **27. Having a platform for sharing emotions and experiences** | 5 4 3 2 1 | 5 4 3 2 1 |
