# Supplementary material for: Analysis of care needs for primary caregivers of premature infants at home based on the Kano model: A cross-sectional study
Source: PLoS One. 2026 Oct 8;21(10):e0360393. doi: 10.1371/journal.pone.0360393 (PMC13648993; doi:10.1371/journal.pone.0360393)
Supplement: Supplementary file 2 [file pone.0360393.s004.docx]

**Response to Reviewers**

**Dear Editor and Reviewers,**

**Thank you for your thoughtful and constructive comments on our manuscript.We have carefully addressed all concerns raised by the Academic Editor and Reviewer. Below we provide a point-by-point response. All revisions have been marked in the revised manuscript with track changes.**

**The main corrections in the paper and respond to the reviewer’s comments are as following:**

****Journal Requirements:****

**When submitting your revision, we need you to address these additional requirements.**

**1.Please ensure that your manuscript meets PLOS ONE's style requirements, including those for file naming.**

****Response：**Thank you for this reminder. We have reformatted the manuscript according to PLOS ONE's style requirements and named the files as requested.**

**2.PLOS requires an ORCID iD for the corresponding author in Editorial Manager on papers submitted after December 6th, 2016. Please ensure that you have an ORCID iD and that it is validated in Editorial Manager.**

****Response：**We have linked the ORCID iD in Editorial Manager. Thanks.**

**3.Please include captions for your Supporting Information files at the end of your manuscript, and update any in-text citations to match accordingly.**

****Response：**Supporting Information captions have been added at the end of the manuscript, and in-text citations have been updated accordingly. Please see the revised manuscript.Thanks.**

**4.If the reviewer comments include a recommendation to cite specific previously published works, please review and evaluate these publications to determine whether they are relevant and should be cited.**

****Response：**The reviewer did not recommend any specific publications for citation. Therefore, no additional references have been added based on this requirement.**

**5. Please review your reference list to ensure that it is complete and correct.**

****Response：** We have reviewed the reference list and confirmed that it is complete and correct, with no retracted articles cited. Two new references have been added to support the revised manuscript.**

****Response to Academic Editor：****

**Comment 1: In a separate file, please copy and paste all of the reviewer critiques, your reply to each of the critiques (line-by-line) with changes to the manuscript and the associated line/section, so that it is easy to follow and read.**

****Response:** We have prepared this Response to Reviewers file accordingly. Below we address each critique line-by-line.**

**Comment 2 (Data availability): The PLOS Data policy requires authors to make all data underlying the findings described in their manuscript fully available without restriction.**

****Response:** Thank you for this reminder. In accordance with the PLOS Data policy, we have provided the anonymized dataset underlying the findings as a Supporting Information file (S1 Data). A Data Availability Statement has also been added at the end of the manuscript, stating that all relevant data are within the manuscript and its Supporting Information files.**

**Comment 3 (Discussion Section): Please provide more context on how your study's results relate to the current literature on caregiver's needs of preterm infants. Currently, this section is modest and does not adequately relate to other studies.**

****Response:** Thank you for this suggestion. We have substantially expanded the Discussion section as follows: (1) in the discussion on the overall level of needs, we added comparisons with relevant literature; (2) in the discussion on survival needs, we added comparisons with previous studies and supplemented with a cross-cultural discussion on the accessibility of first-aid education; and (3) added a new paragraph summarizing what this study adds to existing research. Please see the revised manuscript for details.**

**Comment 4 (Conclusion Section): Please revise this section to state the "take home" message from the results of your study. What would be helpful for the clinician or researcher to know about caregivers needs/preferences, etc. What are some future steps for other studies?**

****Response:** Thank you for this suggestion. The Conclusion section has been rewritten as requested, clarifying the take-home message, practical implications, and future research directions. Please see the revised manuscript.**

****Response to Reviewer ：****

**We thank Reviewer for the thorough and insightful comments. Below we address each point.**

****Abstract****

**Comment 1：Line 27 Should state up to 6 months post discharge**

****Response:** Thank you for this suggestion. We have revised the abstract accordingly to specify "up to 6 months post discharge".**

**Comment 2：Methods should have some comment about the data analysis plan**

****Response:**Thank you for your valuable suggestion. We have revised the Methods section of the Abstract to include some comment about the data analysis plan.**

****Methods：****

**Comment 3：Need information regarding NICU acuity. Does this unit go from extreme GA upwards? Surgical site? Delivery site?**

****Response:**Response: Thank you for these questions. We have added the gestational age range and surgical care information to the Methods section. Regarding the delivery site: our NICU is not a delivery site. Infants are delivered in the delivery room or operating room and then transferred to the NICU as needed.**

**Comment 4 (Lines 113-127 – Exclusion criteria split): The layout of having infant and caregiver inclusion criteria was good. For infant exclusion it seems to be split line 114 and then more at line 126. Also, there should be a full list of exclusion conditions somewhere.**

****Response:** Thank you for pointing this out. The exclusion criteria at line 126 were for primary caregivers, but the incomplete heading caused confusion. We have added the missing headings to clearly separate the exclusion criteria for preterm infants and primary caregivers.**

**Comment 5 (Line 140 – ERG needs): what is ERG needs?**

****Response:** Thank you for pointing this out. We have added the full term in the text: ERG stands for Existence, Relatedness, and Growth needs.**

**Comment 6 (Line 145 – intermediate or senior professional): please define intermediate or senior professional**

****Response:** Thank you for pointing this out. We have added the definition in the text: intermediate or senior professional titles refer to nurse-in-charge or above (including associate chief nurse and chief nurse).**

**Comment 7 (Line 148 – pretest): explain how the pretest was conducted? Or was this the 21 interviews that were described on line 144? Please clarify this section**

****Response:** Thank you for pointing this out. The pretest was conducted after the expert interviews. We have clarified that 30 eligible primary caregivers participated in the pretest, and items were revised based on their feedback until they were easily understood.**

**Comment 8 (Line 201 and line 212 – “double-entered”): what does “double-entered” mean?**

****Response:** Thank you for this question. "Double-entered" means that data were independently entered by two researchers and then cross-verified. We have revised the description to make this clearer.**

**Comment 9 (Questionnaire as appendix): The questions should be an appendix**

****Response:** Thank you for this question.The full questionnaire has been provided as Supporting Information S1 File.**

****Results****

**Comment 10 (Qualified infants during survey time frame): During the survey time frame how many infants would have qualified?**

****Response:** Thank you for this question.We have added that 125 preterm infants were eligible during the survey period, of which 98 were included in the final analysis.**

**Comment 11 (Line 226 – “valid”): Line226 what is meant by valid?**

****Response:** Thank you for this question. We have added the definition of "valid" in the text: valid responses were defined as questionnaires with no missing data on the 27 Kano items and no contradictory responses (e.g., "Like" for both positive and negative forms of the same item).**

**Comment 12 (GA at birth – effect of extreme prematurity): Did the results vary if you factored in GA at birth as more extreme premature infants might have different issues post discharge? I note that the majority of the participants had an infant 34-37 weeks, was the proportion of infants in each GA group consistent with the demographics of the unit?**

****Response:** Thank you for this suggestion. We considered conducting subgroup analysis by gestational age. However, the sample size of extremely and very preterm infants in this study was only 6 cases (6% of the total sample), which is insufficient for stable statistical comparisons. Therefore, we did not perform stratification by gestational age. We have added a statement about this limitation in the Discussion section. Regarding representativeness,the unit's gestational age distribution during the survey period was: 28-31⁺⁶ weeks (8%), 32-33⁺⁶ weeks (40%), and 34-36⁺⁶ weeks (52%). Our sample distribution (6%, 44%, 50%) was generally consistent, indicating good representativeness.**

**Comment 13 (Effect of time since discharge): Did the survey outcomes vary if they were discharged closer to survey date then those further? Example the concerns for a one month versus a 6 month post discharge might be different**

****Response:** Thank you for this insightful suggestion. We agree that analyzing caregiver needs by time since discharge could reveal important dynamic patterns. However, the sample distribution across post‑discharge periods was uneven in our study, and these subgroup sample sizes were too small to support stable statistical comparisons. Therefore, we did not perform such stratification and have added this limitation explicitly in the Discussion section, while also recommending future longitudinal studies to examine the temporal evolution of caregiver needs.**

**Comment 14 (Table 1 – GA and weight ranges): Table 1 – the GA and weight ranges only the first number is listed for example it is 1- not 1-1.4 kg**

****Response:** Thank you for pointing this out. The ranges for gestational age, birth weight, time since discharge, and caregiver age in Table 1 have all been completed.**

**Comment 15 (Table 2 – classification confusing): Table 2 – I find the classification confusing. Maybe a legend might help?**

****Response:** Thank you for this suggestion. We have added a legend below Table 2 to clarify the meanings of A, O, M, and I.**

****Discussion****

**Comment 16 (Closed management system explanation): The discussion mentioned a closed management system. This needs more explanation. It would be helpful in the methods to explain the NICU dynamic and expected role of the caregiver during and towards the end of the admission to prepare for discharge.**

****Response:** Thank you for this suggestion. We have added an explanation of the closed management system in the Methods section: during hospitalization, caregivers are not involved in daily care, and health education is provided at discharge. We also noted that this system is standard in most NICUs in China.**

**Comment 17 (What this adds to current research): Would suggest a section denoting what this adds to the current body of research on the topic**

****Response:** Thank you for this suggestion. A new section titled "What this study adds to existing research" has been added to the Discussion. Please see the revised manuscript.**

****Summary of Changes Made****

**We have uploaded two versions of the revised manuscript: Revised Manuscript with Track Changes – showing all modifications Manuscript – clean version without track changes .**

****Additional correction:** During the revision, we identified and corrected a labeling error in Fig1. One of the data points labeled as "22" has been corrected to "24". The corrected figure has been re-uploaded to the submission system. We apologize for this oversight.**

**Additionally, in response to the technical check, we have now added a complete reference list at the end of the manuscript. The references are numbered in the order of first appearance in the text (1., 2., 3., …), formatted according to PLOS ONE’s Vancouver style, with DOIs included and non-English references marked by language identifiers (e.g., “Chinese.”)**

**Sincerely,**

**Jiaohua Yu**
