# Supplementary material for: Analysis of care needs for primary caregivers of premature infants at home based on the Kano model: A cross-sectional study
Source: PLoS One. 2026 Oct 8;21(10):e0360393. doi: 10.1371/journal.pone.0360393 (PMC13648993; doi:10.1371/journal.pone.0360393)
Supplement: Supplementary file 3 [file pone.0360393.s005.doc]

**Response to Reviewers**

Dear Editor and Reviewers,

We sincerely appreciate the editor and reviewer for their valuable and constructive feedback on our manuscript. We have addressed all the points raised by the Academic Editor and Reviewer. A point-by-point response is provided below. All changes have been highlighted in the revised manuscript using track changes.

The main corrections in the paper and responses to the reviewer's comments are as follows:

**Journal Requirements**

1. If the reviewer comments include a recommendation to cite specific previously published works, please review and evaluate these publications to determine whether they are relevant and should be cited.

Response: The reviewer did not recommend any specific publications for citation. Therefore, no additional references have been added based on this requirement.

2. Please review your reference list to ensure that it is complete and correct.

Response: We have reviewed the reference list and confirmed that it is complete and correct, with no retracted articles cited. The references remain unchanged in this revision.

**Response to Reviewer #1**

We thank Reviewer #1 for the thorough and insightful comments. Below we address each point.

**Comment 1: Line 51 – 6th for what? lowest rate?**

**Response:** We appreciate the reviewer’s question. The “6%” refers to the preterm birth rate in China, while “ranking fourth globally” refers to the absolute number of preterm births. According to the cited reference (Ohuma et al., 2023), the global preterm birth rate was approximately 9.9%, whereas China’s preterm birth rate was approximately 6.1% . However, the absolute number of preterm births in China ranked fourth globally. We have revised the sentence to make this distinction clear. The revised sentence now reads: “In China, the preterm birth rate is approximately 6%, while the number of preterm births ranks fourth globally [1]” **(please refer to page 3, lines 51–52).**

**Comment 2: Line 123 – what were the severe conditions that would exclude an infant?**

Response: Thank you for pointing this out. We agree that the original description was not specific enough. We have now listed the severe complications explicitly and defined their severity where applicable. The revised sentence reads: “(1) severe complications including moderate-to-severe anemia, severe retinopathy of prematurity requiring treatment, severe intraventricular hemorrhage, necrotizing enterocolitis requiring surgery, and moderate-to-severe bronchopulmonary dysplasia”(**please refer to page 6 , lines 124-126)**

**Comment 3: Line 126 – why was there an age difference between mothers and fathers in the inclusion criteria?**

**Response:** We are grateful for this comment. The age criteria were pre-specified at the study design stage. The target population of this study was typical parent caregivers of preterm infants, and the age criteria were set with reference to the legal marriage age in China (female ≥20 years, male ≥22 years) [16]. Caregivers below this age were not included because this study focused on adult caregivers at the life stage typical of parent caregivers in the Chinese context; these caregivers may differ in social roles, support systems, and caregiving experience, which could introduce heterogeneity into the sample. The criterion involved age only. Grandparent caregivers were older and automatically met this criterion. We have clarified this in the revised manuscript. The revised sentence reads: “(1) adult primary caregivers, males ≥22 years old, females ≥20 years old [16]”(**please refer to page 7 , lines 129-130)**

**Comment 4: Line 130 – why is there health conditions of primary caregivers that makes them excluded?**

**Response:** We appreciate this important point. The exclusion criterion was originally intended to exclude caregivers whose caregiving ability was impaired by severe cardiac, cerebral, pulmonary, or renal diseases, but the original wording did not make this clear. We have now described the criterion more specifically to clarify the rationale: such conditions may compromise the caregiver's ability to provide care and could confound the assessment of care needs. The revised sentence reads: “(1) caregivers whose caregiving ability was impaired by severe cardiac, cerebral, pulmonary, or renal diseases; and (2) caregivers who withdrew during the study.”(**please refer to page 7 , lines 133-135)**

**Comment 5: Line 233 – do not need the last sentence it was already explained above.**

**Response:** We thank the reviewer for this suggestion. We have removed the last sentence in the Ethical Considerations section (“Participants were informed of the study’s purpose and their voluntary participation prior to data collection.”), as it was redundant with the Quality Control section, which already states that participants were informed of the voluntary, anonymous, and confidential nature of the study.

**Summary of Changes Made**

We have uploaded two versions of the revised manuscript:

Revised Manuscript with Track Changes – showing all modifications

Manuscript – clean version without track changes

**Additional Note**

We would also like to note that during the revision, we have updated the affiliations of authors Fenfen Lan, Zhaohua Huo, Yuanyuan Wang, Qiaoyun Shi, and Juan Liu to reflect the official institutional name change of the authors' institution. The new affiliation is: Affiliated Central People's Hospital, China Three Gorges University (Yichang Central People's Hospital). This change has been made in both the manuscript (**page 1**) and the online submission system. The authorship order and all author names remain unchanged. We confirm that this update reflects the correct institutional name as required by our hospital administration.

We are grateful for the reviewers' and editors' thoughtful and constructive feedback, which has significantly improved the quality of our manuscript. We hope the revised manuscript now meets the publication criteria of PLOS ONE. Thank you again for your time and consideration.

Sincerely,

Jiaohua Yu
