# Supplementary material for: Analysis of care needs for primary caregivers of premature infants at home based on the Kano model: A cross-sectional study
Source: PLoS One. 2026 Oct 8;21(10):e0360393. doi: 10.1371/journal.pone.0360393 (PMC13648993; doi:10.1371/journal.pone.0360393)
Supplement: Peer review history

# Peer review: Decision Letter 0

**DOI:** 10.1371/journal.pone.0360393.r001

**Authors:** Nhu Tran

15 Apr 2026

Dear Dr. yu,

Thank you for submitting your manuscript to PLOS ONE. After careful consideration, we feel that it has merit but does not fully meet PLOS ONE’s publication criteria as it currently stands. Therefore, we invite you to submit a revised version of the manuscript that addresses the points raised during the review process.

==============================

Thank you for your patience in the review process.

1) In a separate file, please copy and paste all of the reviewer critiques, your reply to each of the critiques (line-by-line) with changes to the manuscript and the associated line/section, so that it is easy to follow and read.

2) The PLOS Data policy requires authors to make all data underlying the findings described in their manuscript fully available without restriction, with rare exception (please refer to the Data Availability Statement in the manuscript PDF file). The data should be provided as part of the manuscript or its supporting information, or deposited to a public repository. For example, in addition to summary statistics, the data points behind means, medians and variance measures should be available. If there are restrictions on publicly sharing data—e.g. participant privacy or use of data from a third party—those must be specified.

3) Discussion Section: Please provide more context on how your study's results relate to the current literature on caregiver's needs of preterm infants. Currently, this section is modest and does not adequately relate to other studies.

4) Conclusion Section: Please revise this section to state the "take home" message from the results of your study. What would be helpful for the clinician or researcher to know about caregivers needs/preferences, etc. What are some future steps for other studies?

==============================

Please submit your revised manuscript by May 30 2026 11:59PM. If you will need more time than this to complete your revisions, please reply to this message or contact the journal office at plosone@plos.org. When you're ready to submit your revision, log on to https://www.editorialmanager.com/pone/ and select the 'Submissions Needing Revision' folder to locate your manuscript file.

Please include the following items when submitting your revised manuscript:

- A letter that responds to each point raised by the academic editor and reviewer(s). You should upload this letter as a separate file labeled 'Response to Reviewers'.
- A marked-up copy of your manuscript that highlights changes made to the original version. You should upload this as a separate file labeled 'Revised Manuscript with Track Changes'.
- An unmarked version of your revised paper without tracked changes. You should upload this as a separate file labeled 'Manuscript'.

If you would like to make changes to your financial disclosure, please include your updated statement in your cover letter. Guidelines for resubmitting your figure files are available below the reviewer comments at the end of this letter.

If applicable, we recommend that you deposit your laboratory protocols in protocols.io to enhance the reproducibility of your results. Protocols.io assigns your protocol its own identifier (DOI) so that it can be cited independently in the future. For instructions see: https://journals.plos.org/plosone/s/submission-guidelines#loc-laboratory-protocols. Additionally, PLOS ONE offers an option for publishing peer-reviewed Lab Protocol articles, which describe protocols hosted on protocols.io. Read more information on sharing protocols at https://plos.org/protocols?utm_medium=editorial-email&utm_source=authorletters&utm_campaign=protocols.

As the corresponding author, your ORCID iD is verified in the submission system and will appear in the published article. PLOS supports the use of ORCID, and we encourage all coauthors to register for an ORCID iD and use it as well. Please encourage your coauthors to verify their ORCID iD within the submission system before final acceptance, as unverified ORCID iDs will not appear in the published article. *Only*  the individual author can complete the verification step; PLOS staff *cannot*  verify ORCID iDs on behalf of authors.

We look forward to receiving your revised manuscript.

Kind regards,

Nhu N. Tran

Academic Editor

PLOS One

Journal Requirements:

When submitting your revision, we need you to address these additional requirements.

1. Please ensure that your manuscript meets PLOS ONE's style requirements, including those for file naming. The PLOS ONE style templates can be found at

https://journals.plos.org/plosone/s/file?id=wjVg/PLOSOne_formatting_sample_main_body.pdf and

https://journals.plos.org/plosone/s/file?id=ba62/PLOSOne_formatting_sample_title_authors_affiliations.pdf

2. PLOS requires an ORCID iD for the corresponding author in Editorial Manager on papers submitted after December 6th, 2016. Please ensure that you have an ORCID iD and that it is validated in Editorial Manager. To do this, go to ‘Update my Information’ (in the upper left-hand corner of the main menu), and click on the Fetch/Validate link next to the ORCID field. This will take you to the ORCID site and allow you to create a new iD or authenticate a pre-existing iD in Editorial Manager.

3. Please include captions for your Supporting Information files at the end of your manuscript, and update any in-text citations to match accordingly. Please see our Supporting Information guidelines for more information: http://journals.plos.org/plosone/s/supporting-information.

4. If the reviewer comments include a recommendation to cite specific previously published works, please review and evaluate these publications to determine whether they are relevant and should be cited. There is no requirement to cite these works unless the editor has indicated otherwise.

5. Please review your reference list to ensure that it is complete and correct. If you have cited papers that have been retracted, please include the rationale for doing so in the manuscript text, or remove these references and replace them with relevant current references. Any changes to the reference list should be mentioned in the rebuttal letter that accompanies your revised manuscript. If you need to cite a retracted article, indicate the article’s retracted status in the References list and also include a citation and full reference for the retraction notice.

[Note: HTML markup is below. Please do not edit.]

Reviewers' comments:

Reviewer's Responses to Questions

**Comments to the Author**

1. Is the manuscript technically sound, and do the data support the conclusions?

Reviewer #1: Yes

**********

2. Has the statistical analysis been performed appropriately and rigorously?

Reviewer #1: Yes

**********

3. Have the authors made all data underlying the findings in their manuscript fully available?

The PLOS Data policy

Reviewer #1: No

**********

4. Is the manuscript presented in an intelligible fashion and written in standard English?

Reviewer #1: Yes

**********

Reviewer #1: Abstract

• Line 27 Should state up to 6 months post discharge

• Methods should have some comment about the data analysis plan

Methods

• Need information regarding NICU acuity. Does this unit go from extreme GA upwards? Surgical site? Delivery site?

• Line 113-127. The layout of having infant and caregiver inclusion criteria was good. For infant exclusion it seems to be split line 114 and then more at line 126. Also, there should be a full list of exclusion conditions somewhere

• Line 140 what is ERG needs?

• Line 145 please define intermediate or senior professional

• Line 148 explain how the pretest was conducted? Or was this the 21 interviews that were described on line 144? Please clarify this section

• Line 201 and again on line 212 what does “double-entered” mean?

• The questions should be an appendix

Results

• During the survey time frame how many infants would have qualified?

• Line226 what is meant by valid?

• Did the results vary if you factored in GA at birth as more extreme premature infants might have different issues post discharge? I note that the majority of the participants had an infant 34-37 weeks, was the proportion of infants in each GA group consistent with the demographics of the unit?

• Did the survey outcomes vary if they were discharged closer to survey date then those further? Example the concerns for a one month versus a 6 month post discharge might be different

• Table 1 – the GA and weight ranges only the first number is listed for example it is 1- not 1-1.4 kg

• Table 2 – I find the classification confusing. Maybe a legend might help?

Discussion

• The discussion mentioned a closed management system. This needs more explanation. It would be helpful in the methods to explain the NICU dynamic and expected role of the caregiver during and towards the end of the admission to prepare for discharge.

• Would suggest a section denoting what this adds to the current body of research on the topic

**********

what does this mean?). If published, this will include your full peer review and any attached files.

If you choose “no”, your identity will remain anonymous but your review may still be made public.

**Do you want your identity to be public for this peer review?**  For information about this choice, including consent withdrawal, please see our Privacy Policy

Reviewer #1: No

**********

[NOTE: If reviewer comments were submitted as an attachment file, they will be attached to this email and accessible via the submission site. Please log into your account, locate the manuscript record, and check for the action link "View Attachments". If this link does not appear, there are no attachment files.]

To ensure your figures meet our technical requirements, please review our figure guidelines: https://journals.plos.org/plosone/s/figures

You may also use PLOS’s free figure tool, NAAS, to help you prepare publication quality figures: https://journals.plos.org/plosone/s/figures#loc-tools-for-figure-preparation.

NAAS will assess whether your figures meet our technical requirements by comparing each figure against our figure specifications.

# Author comment: Author response to Decision Letter 1

**DOI:** 10.1371/journal.pone.0360393.r002

13 May 2026

Dear Editor and Reviewers,

Thank you for your thoughtful and constructive comments on our manuscript.We have carefully addressed all concerns raised by the Academic Editor and Reviewer. Below we provide a point-by-point response. All revisions have been marked in the revised manuscript with track changes.

The main corrections in the paper and respond to the reviewer’s comments are as following:

Journal Requirements:

When submitting your revision, we need you to address these additional requirements.

1.Please ensure that your manuscript meets PLOS ONE's style requirements, including those for file naming.

Response：Thank you for this reminder. We have reformatted the manuscript according to PLOS ONE's style requirements and named the files as requested.

2.PLOS requires an ORCID iD for the corresponding author in Editorial Manager on papers submitted after December 6th, 2016. Please ensure that you have an ORCID iD and that it is validated in Editorial Manager.

Response：We have linked the ORCID iD in Editorial Manager. Thanks.

3.Please include captions for your Supporting Information files at the end of your manuscript, and update any in-text citations to match accordingly.

Response：Supporting Information captions have been added at the end of the manuscript, and in-text citations have been updated accordingly. Please see the revised manuscript.Thanks.

4.If the reviewer comments include a recommendation to cite specific previously published works, please review and evaluate these publications to determine whether they are relevant and should be cited.

Response：The reviewer did not recommend any specific publications for citation. Therefore, no additional references have been added based on this requirement.

5. Please review your reference list to ensure that it is complete and correct.

Response： We have reviewed the reference list and confirmed that it is complete and correct, with no retracted articles cited. Two new references have been added to support the revised manuscript.

Response to Academic Editor：

Comment 1: In a separate file, please copy and paste all of the reviewer critiques, your reply to each of the critiques (line-by-line) with changes to the manuscript and the associated line/section, so that it is easy to follow and read.

Response: We have prepared this Response to Reviewers file accordingly. Below we address each critique line-by-line.

Comment 2 (Data availability): The PLOS Data policy requires authors to make all data underlying the findings described in their manuscript fully available without restriction.

Response: Thank you for this reminder. In accordance with the PLOS Data policy, we have provided the anonymized dataset underlying the findings as a Supporting Information file (S1 Data). A Data Availability Statement has also been added at the end of the manuscript, stating that all relevant data are within the manuscript and its Supporting Information files.

Comment 3 (Discussion Section): Please provide more context on how your study's results relate to the current literature on caregiver's needs of preterm infants. Currently, this section is modest and does not adequately relate to other studies.

Response: Thank you for this suggestion. We have substantially expanded the Discussion section as follows: (1) in the discussion on the overall level of needs, we added comparisons with relevant literature; (2) in the discussion on survival needs, we added comparisons with previous studies and supplemented with a cross-cultural discussion on the accessibility of first-aid education; and (3) added a new paragraph summarizing what this study adds to existing research. Please see the revised manuscript for details.

Comment 4 (Conclusion Section): Please revise this section to state the "take home" message from the results of your study. What would be helpful for the clinician or researcher to know about caregivers needs/preferences, etc. What are some future steps for other studies?

Response: Thank you for this suggestion. The Conclusion section has been rewritten as requested, clarifying the take-home message, practical implications, and future research directions. Please see the revised manuscript.

Response to Reviewer ：

We thank Reviewer for the thorough and insightful comments. Below we address each point.

Abstract

Comment 1：Line 27 Should state up to 6 months post discharge

Response: Thank you for this suggestion. We have revised the abstract accordingly to specify "up to 6 months post discharge".

Comment 2：Methods should have some comment about the data analysis plan

Response:Thank you for your valuable suggestion. We have revised the Methods section of the Abstract to include some comment about the data analysis plan.

Methods：

Comment 3：Need information regarding NICU acuity. Does this unit go from extreme GA upwards? Surgical site? Delivery site?

Response:Response: Thank you for these questions. We have added the gestational age range and surgical care information to the Methods section. Regarding the delivery site: our NICU is not a delivery site. Infants are delivered in the delivery room or operating room and then transferred to the NICU as needed.

Comment 4 (Lines 113-127 – Exclusion criteria split): The layout of having infant and caregiver inclusion criteria was good. For infant exclusion it seems to be split line 114 and then more at line 126. Also, there should be a full list of exclusion conditions somewhere.

Response: Thank you for pointing this out. The exclusion criteria at line 126 were for primary caregivers, but the incomplete heading caused confusion. We have added the missing headings to clearly separate the exclusion criteria for preterm infants and primary caregivers.

Comment 5 (Line 140 – ERG needs): what is ERG needs?

Response: Thank you for pointing this out. We have added the full term in the text: ERG stands for Existence, Relatedness, and Growth needs.

Comment 6 (Line 145 – intermediate or senior professional): please define intermediate or senior professional

Response: Thank you for pointing this out. We have added the definition in the text: intermediate or senior professional titles refer to nurse-in-charge or above (including associate chief nurse and chief nurse).

Comment 7 (Line 148 – pretest): explain how the pretest was conducted? Or was this the 21 interviews that were described on line 144? Please clarify this section

Response: Thank you for pointing this out. The pretest was conducted after the expert interviews. We have clarified that 30 eligible primary caregivers participated in the pretest, and items were revised based on their feedback until they were easily understood.

Comment 8 (Line 201 and line 212 – “double-entered”): what does “double-entered” mean?

Response: Thank you for this question. "Double-entered" means that data were independently entered by two researchers and then cross-verified. We have revised the description to make this clearer.

Comment 9 (Questionnaire as appendix): The questions should be an appendix

Response: Thank you for this question.The full questionnaire has been provided as Supporting Information S1 File.

Results

Comment 10 (Qualified infants during survey time frame): During the survey time frame how many infants would have qualified?

Response: Thank you for this question.We have added that 125 preterm infants were eligible during the survey period, of which 98 were included in the final analysis.

Comment 11 (Line 226 – “valid”): Line226 what is meant by valid?

Response: Thank you for this question. We have added the definition of "valid" in the text: valid responses were defined as questionnaires with no missing data on the 27 Kano items and no contradictory responses (e.g., "Like" for both positive and negative forms of the same item).

Comment 12 (GA at birth – effect of extreme prematurity): Did the results vary if you factored in GA at birth as more extreme premature infants might have different issues post discharge? I note that the majority of the participants had an infant 34-37 weeks, was the proportion of infants in each GA group consistent with the demographics of the unit?

Response: Thank you for this suggestion. We considered conducting subgroup analysis by gestational age. However, the sample size of extremely and very preterm infants in this study was only 6 cases (6% of the total sample), which is insufficient for stable statistical comparisons. Therefore, we did not perform stratification by gestational age. We have added a statement about this limitation in the Discussion section. Regarding representativeness,the unit's gestational age distribution during the survey period was: 28-31⁺⁶ weeks (8%), 32-33⁺⁶ weeks (40%), and 34-36⁺⁶ weeks (52%). Our sample distribution (6%, 44%, 50%) was generally consistent, indicating good representativeness.

Comment 13 (Effect of time since discharge): Did the survey outcomes vary if they were discharged closer to survey date then those further? Example the concerns for a one month versus a 6 month post discharge might be different

Response: Thank you for this insightful suggestion. We agree that analyzing caregiver needs by time since discharge could reveal important dynamic patterns. However, the sample distribution across post‑discharge periods was uneven in our study, and these subgroup sample sizes were too small to support stable statistical comparisons. Therefore, we did not perform such stratification and have added this limitation explicitly in the Discussion section, while also recommending future longitudinal studies to examine the temporal evolution of caregiver needs.

Comment 14 (Table 1 – GA and weight ranges): Table 1 – the GA and weight ranges only the first number is listed for example it is 1- not 1-1.4 kg

Response: Thank you for pointing this out. The ranges for gestational age, birth weight, time since discharge, and caregiver age in Table 1 have all been completed.

Comment 15 (Table 2 – classification confusing): Table 2 – I find the classification confusing. Maybe a legend might help?

Response: Thank you for this suggestion. We have added a legend below Table 2 to clarify the meanings of A, O, M, and I.

Discussion

Comment 16 (Closed management system explanation): The discussion mentioned a closed management system. This needs more explanation. It would be helpful in the methods to explain the NICU dynamic and expected role of the caregiver during and towards the end of the admission to prepare for discharge.

Response: Thank you for this suggestion. We have added an explanation of the closed management system in the Methods section: during hospitalization, caregivers are not involved in daily care, and health education is provided at discharge. We also noted that this system is standard in most NICUs in China.

Comment 17 (What this adds to current research): Would suggest a section denoting what this adds to the current body of research on the topic

Response: Thank you for this suggestion. A new section titled "What this study adds to existing research" has been added to the Discussion. Please see the revised manuscript.

Summary of Changes Made

We have uploaded two versions of the revised manuscript: Revised Manuscript with Track Changes – showing all modifications Manuscript – clean version without track changes .

Additional correction: During the revision, we identified and corrected a labeling error in Fig1. One of the data points labeled as "22" has been corrected to "24". The corrected figure has been re-uploaded to the submission system. We apologize for this oversight.

Additionally, in response to the technical check, we have now added a complete reference list at the end of the manuscript. The references are numbered in the order of first appearance in the text (1., 2., 3., …), formatted according to PLOS ONE’s Vancouver style, with DOIs included and non-English references marked by language identifiers (e.g., “Chinese.”)

Sincerely,

Jiaohua Yu

Attachment Submitted filename: Response to Reviewers.docx

# Peer review: Decision Letter 1

**DOI:** 10.1371/journal.pone.0360393.r003

**Authors:** Helen Howard

11 Sep 2026

PONE-D-26-02017R1

Analysis of care needs for primary caregivers of premature infants at home based on the Kano model: A cross-sectional study

PLOS One

Dear Dr. yu,

Thank you for submitting your manuscript to PLOS One. After careful consideration, we feel that it has merit but does not fully meet PLOS One’s publication criteria as it currently stands. Therefore, we invite you to submit a revised version of the manuscript that addresses the points raised during the review process.

Please submit your revised manuscript by Oct 26 2026 11:59PM. If you will need more time than this to complete your revisions, please reply to this message or contact the journal office at plosone@plos.org. When you're ready to submit your revision, log on to https://www.editorialmanager.com/pone/ and select the 'Submissions Needing Revision' folder to locate your manuscript file.

Please include the following items when submitting your revised manuscript:

- A letter that responds to each point raised by the academic editor and reviewer(s). You should upload this letter as a separate file labeled 'Response to Reviewers'.
- A marked-up copy of your manuscript that highlights changes made to the original version. You should upload this as a separate file labeled 'Revised Manuscript with Track Changes'.
- An unmarked version of your revised paper without tracked changes. You should upload this as a separate file labeled 'Manuscript'.

If you would like to make changes to your financial disclosure, please include your updated statement in your cover letter. Guidelines for resubmitting your figure files are available below the reviewer comments at the end of this letter.

If applicable, we recommend that you deposit your laboratory protocols in protocols.io to enhance the reproducibility of your results. Protocols.io assigns your protocol its own identifier (DOI) so that it can be cited independently in the future. For instructions see: https://journals.plos.org/plosone/s/submission-guidelines#loc-laboratory-protocols. Additionally, PLOS One offers an option for publishing peer-reviewed Lab Protocol articles, which describe protocols hosted on protocols.io. Read more information on sharing protocols at https://plos.org/protocols?utm_medium=editorial-email&utm_source=authorletters&utm_campaign=protocols.

As the corresponding author, your ORCID iD is verified in the submission system and will appear in the published article. PLOS supports the use of ORCID, and we encourage all coauthors to register for an ORCID iD and use it as well. Please encourage your coauthors to verify their ORCID iD within the submission system before final acceptance, as unverified ORCID iDs will not appear in the published article. *Only*  the individual author can complete the verification step; PLOS staff *cannot*  verify ORCID iDs on behalf of authors.

We look forward to receiving your revised manuscript.

Kind regards,

Helen Howard

Staff Editor

PLOS One

**Journal Requirements:**

1. If the reviewer comments include a recommendation to cite specific previously published works, please review and evaluate these publications to determine whether they are relevant and should be cited. There is no requirement to cite these works unless the editor has indicated otherwise.

2. Please review your reference list to ensure that it is complete and correct. If you have cited papers that have been retracted, please include the rationale for doing so in the manuscript text, or remove these references and replace them with relevant current references. Any changes to the reference list should be mentioned in the rebuttal letter that accompanies your revised manuscript. If you need to cite a retracted article, indicate the article’s retracted status in the References list and also include a citation and full reference for the retraction notice.

[Note: HTML markup is below. Please do not edit.]

Reviewers' comments:

Reviewer's Responses to Questions

**Comments to the Author**

1. If the authors have adequately addressed your comments raised in a previous round of review and you feel that this manuscript is now acceptable for publication, you may indicate that here to bypass the “Comments to the Author” section, enter your conflict of interest statement in the “Confidential to Editor” section, and submit your "Accept" recommendation.

Reviewer #1: All comments have been addressed

**********

2. Is the manuscript technically sound, and do the data support the conclusions?

The manuscript must describe a technically sound piece of scientific research with data that supports the conclusions. Experiments must have been conducted rigorously, with appropriate controls, replication, and sample sizes. The conclusions must be drawn appropriately based on the data presented.

Reviewer #1: Yes

**********

3. Has the statistical analysis been performed appropriately and rigorously?

Reviewer #1: Yes

**********

4. Have the authors made all data underlying the findings in their manuscript fully available?

The PLOS Data policy requires authors to make all data underlying the findings described in their manuscript fully available without restriction, with rare exception (please refer to the Data Availability Statement in the manuscript PDF file). The data should be provided as part of the manuscript or its supporting information, or deposited to a public repository. For example, in addition to summary statistics, the data points behind means, medians and variance measures should be available. If there are restrictions on publicly sharing data—e.g. participant privacy or use of data from a third party—those must be specified.

Reviewer #1: Yes

**********

5. Is the manuscript presented in an intelligible fashion and written in standard English?

PLOS ONE does not copyedit accepted manuscripts, so the language in submitted articles must be clear, correct, and unambiguous. Any typographical or grammatical errors should be corrected at revision, so please note any specific errors here.

Reviewer #1: Yes

**********

6. Review Comments to the Author

Please use the space provided to explain your answers to the questions above. You may also include additional comments for the author, including concerns about dual publication, research ethics, or publication ethics. (Please upload your review as an attachment if it exceeds 20,000 characters)

Reviewer #1: Comment are from the tracked changes version

• Line 51 – 6th for what? lowest rate?

• Line 123 – what were the severe conditions that would exclude an infant?

• Line 126 why was there an age difference between mothers and fathers in the inclusion criteria?

• Line 130 – why is there health conditions of primary caregivers that makes them excluded?

• Lin3 233 – do not need the last sentence it was already explained above

**********

7. PLOS authors have the option to publish the peer review history of their article (what does this mean?). If published, this will include your full peer review and any attached files.

If you choose “no”, your identity will remain anonymous but your review may still be made public.

**Do you want your identity to be public for this peer review?**  For information about this choice, including consent withdrawal, please see our Privacy Policy.

Reviewer #1: No

**********

[NOTE: If reviewer comments were submitted as an attachment file, they will be attached to this email and accessible via the submission site. Please log into your account, locate the manuscript record, and check for the action link "View Attachments". If this link does not appear, there are no attachment files.]

To ensure your figures meet our technical requirements, please review our figure guidelines: https://journals.plos.org/plosone/s/figures

You may also use PLOS’s free figure tool, NAAS, to help you prepare publication quality figures: https://journals.plos.org/plosone/s/figures#loc-tools-for-figure-preparation.

NAAS will assess whether your figures meet our technical requirements by comparing each figure against our figure specifications.

# Author comment: Author response to Decision Letter 2

**DOI:** 10.1371/journal.pone.0360393.r004

13 Sep 2026

Dear Editor and Reviewers,

We sincerely appreciate the editor and reviewer for their valuable and constructive feedback on our manuscript. We have addressed all the points raised by the Academic Editor and Reviewer. A point-by-point response is provided below. All changes have been highlighted in the revised manuscript using track changes.

The main corrections in the paper and responses to the reviewer's comments are as follows:

Journal Requirements

1. If the reviewer comments include a recommendation to cite specific previously published works, please review and evaluate these publications to determine whether they are relevant and should be cited.

Response: The reviewer did not recommend any specific publications for citation. Therefore, no additional references have been added based on this requirement.

2. Please review your reference list to ensure that it is complete and correct.

Response: We have reviewed the reference list and confirmed that it is complete and correct, with no retracted articles cited. The references remain unchanged in this revision.

Response to Reviewer #1

We thank Reviewer #1 for the thorough and insightful comments. Below we address each point.

Comment 1: Line 51 – 6th for what? lowest rate?

Response: We appreciate the reviewer’s question. The “6%” refers to the preterm birth rate in China, while “ranking fourth globally” refers to the absolute number of preterm births. According to the cited reference (Ohuma et al., 2023), the global preterm birth rate was approximately 9.9%, whereas China’s preterm birth rate was approximately 6.1% . However, the absolute number of preterm births in China ranked fourth globally. We have revised the sentence to make this distinction clear. The revised sentence now reads: “In China, the preterm birth rate is approximately 6%, while the number of preterm births ranks fourth globally [1]” (please refer to page 3, lines 51–52).

Comment 2: Line 123 – what were the severe conditions that would exclude an infant?

Response: Thank you for pointing this out. We agree that the original description was not specific enough. We have now listed the severe complications explicitly and defined their severity where applicable. The revised sentence reads: “(1) severe complications including moderate-to-severe anemia, severe retinopathy of prematurity requiring treatment, severe intraventricular hemorrhage, necrotizing enterocolitis requiring surgery, and moderate-to-severe bronchopulmonary dysplasia”(please refer to page 6 , lines 124-126)

Comment 3: Line 126 – why was there an age difference between mothers and fathers in the inclusion criteria?

Response: We are grateful for this comment. The age criteria were pre-specified at the study design stage. The target population of this study was typical parent caregivers of preterm infants, and the age criteria were set with reference to the legal marriage age in China (female ≥20 years, male ≥22 years) [16]. Caregivers below this age were not included because this study focused on adult caregivers at the life stage typical of parent caregivers in the Chinese context; these caregivers may differ in social roles, support systems, and caregiving experience, which could introduce heterogeneity into the sample. The criterion involved age only. Grandparent caregivers were older and automatically met this criterion. We have clarified this in the revised manuscript. The revised sentence reads: “(1) adult primary caregivers, males ≥22 years old, females ≥20 years old [16]”(please refer to page 7 , lines 129-130)

Comment 4: Line 130 – why is there health conditions of primary caregivers that makes them excluded?

Response: We appreciate this important point. The exclusion criterion was originally intended to exclude caregivers whose caregiving ability was impaired by severe cardiac, cerebral, pulmonary, or renal diseases, but the original wording did not make this clear. We have now described the criterion more specifically to clarify the rationale: such conditions may compromise the caregiver's ability to provide care and could confound the assessment of care needs. The revised sentence reads: “(1) caregivers whose caregiving ability was impaired by severe cardiac, cerebral, pulmonary, or renal diseases; and (2) caregivers who withdrew during the study.”(please refer to page 7 , lines 133-135)

Comment 5: Line 233 – do not need the last sentence it was already explained above.

Response: We thank the reviewer for this suggestion. We have removed the last sentence in the Ethical Considerations section (“Participants were informed of the study’s purpose and their voluntary participation prior to data collection.”), as it was redundant with the Quality Control section, which already states that participants were informed of the voluntary, anonymous, and confidential nature of the study.

Summary of Changes Made

We have uploaded two versions of the revised manuscript:

Revised Manuscript with Track Changes – showing all modifications

Manuscript – clean version without track changes

Additional Note

We would also like to note that during the revision, we have updated the affiliations of authors Fenfen Lan, Zhaohua Huo, Yuanyuan Wang, Qiaoyun Shi, and Juan Liu to reflect the official institutional name change of our hospital. The new affiliation is: Affiliated Central People's Hospital, China Three Gorges University (Yichang Central People's Hospital). This change has been made in both the title page(page 1) and the online submission system. The authorship order and all author names remain unchanged. We confirm that this update reflects the correct institutional name as required by our hospital administration.

We are grateful for the reviewers' and editors' thoughtful and constructive feedback, which has significantly improved the quality of our manuscript. We hope the revised manuscript now meets the publication criteria of PLOS ONE. Thank you again for your time and consideration.

Sincerely,

Jiaohua Yu

Attachment Submitted filename: Response to Reviewers.doc

# Peer review: Decision Letter 2

**DOI:** 10.1371/journal.pone.0360393.r005

**Authors:** Jing He

28 Sep 2026

Analysis of care needs for primary caregivers of premature infants at home based on the Kano model: A cross-sectional study

PONE-D-26-02017R2

Dear Prof. Yu,

We’re pleased to inform you that your manuscript has been judged scientifically suitable for publication and will be formally accepted for publication once it meets all outstanding technical requirements.

Within one week, you’ll receive an e-mail detailing the required amendments. When these have been addressed, you’ll receive a formal acceptance letter and your manuscript will be scheduled for publication.

An invoice will be generated when your article is formally accepted. Please note, if your institution has a publishing partnership with PLOS and your article meets the relevant criteria, all or part of your publication costs will be covered. Please make sure your user information is up-to-date by logging into Editorial Manager at Editorial Manager® and clicking the ‘Update My Information' link at the top of the page. For questions related to billing, please contact billing support.

If your institution or institutions have a press office, please notify them about your upcoming paper to help maximize its impact. If they’ll be preparing press materials, please inform our press team as soon as possible -- no later than 48 hours after receiving the formal acceptance. Your manuscript will remain under strict press embargo until 2 pm Eastern Time on the date of publication. For more information, please contact onepress@plos.org.

Kind regards,

Jing He

Academic Editor

PLOS One

Additional Editor Comments (optional):

The authors have satisfactorily addressed all the reviewers' comments. We are pleased to inform you that your manuscript has been accepted for publication.

Reviewers' comments:

Reviewer's Responses to Questions

**Comments to the Author**

Reviewer #1: All comments have been addressed

**********

2. Is the manuscript technically sound, and do the data support the conclusions?

Reviewer #1: Yes

**********

3. Has the statistical analysis been performed appropriately and rigorously?

Reviewer #1: Yes

**********

4. Have the authors made all data underlying the findings in their manuscript fully available?

The PLOS Data policy

Reviewer #1: Yes

**********

5. Is the manuscript presented in an intelligible fashion and written in standard English?

Reviewer #1: Yes

**********

Reviewer #1: (No Response)

**********

what does this mean?). If published, this will include your full peer review and any attached files.

If you choose “no”, your identity will remain anonymous but your review may still be made public.

**Do you want your identity to be public for this peer review?**  For information about this choice, including consent withdrawal, please see our Privacy Policy

Reviewer #1: No

**********

# Editor report: Acceptance letter

**DOI:** 10.1371/journal.pone.0360393.r006

**Authors:** Jing He

PONE-D-26-02017R2

PLOS One

Dear Dr. yu,

I'm pleased to inform you that your manuscript has been deemed suitable for publication in PLOS One. Congratulations! Your manuscript is now being handed over to our production team.

At this stage, our production department will prepare your paper for publication. This includes ensuring the following:

* All references, tables, and figures are properly cited

* All relevant supporting information is included in the manuscript submission,

* There are no issues that prevent the paper from being properly typeset

You will receive further instructions from the production team, including instructions on how to review your proof when it is ready. Please keep in mind that we are working through a large volume of accepted articles, so please give us a few days to review your paper and let you know the next and final steps.

Lastly, if your institution or institutions have a press office, please let them know about your upcoming paper now to help maximize its impact. If they'll be preparing press materials, please inform our press team within the next 48 hours. Your manuscript will remain under strict press embargo until 2 pm Eastern Time on the date of publication. For more information, please contact onepress@plos.org.

You will receive an invoice from PLOS for your publication fee after your manuscript has reached the completed accept phase. If you receive an email requesting payment before acceptance or for any other service, this may be a phishing scheme. Learn how to identify phishing emails and protect your accounts at https://explore.plos.org/phishing.

If we can help with anything else, please email us at customercare@plos.org.

Thank you for submitting your work to PLOS One and supporting open access.

Kind regards,

PLOS One Editorial Office Staff

on behalf of

Dr. Jing He

Academic Editor

PLOS One
